# Safety and feasibility of toripalimab plus lenvatinib with or without radiotherapy in advanced BTC

**DOI:** 10.3389/fimmu.2023.1084843

**Published:** 2023-01-17

**Authors:** Yunchao Wang, Nan Zhang, Jingnan Xue, Chengpei Zhu, Yanyu Wang, Longhao Zhang, Xu Yang, Hao Wang, Shanshan Wang, Jiashuo Chao, Xiaobo Yang, Haitao Zhao

**Affiliations:** Department of Liver Surgery, Peking Union Medical College Hospital, Chinese Academy of Medical Sciences and Peking Union Medical College (CAMS & PUMC), Beijing, China

**Keywords:** advanced biliary tract cancer, PD-1 inhibitor, lenvatinib, radiotherapy, synergic effect

## Abstract

**Background:**

Toripalimab shows antitumor efficacy in cholangiocarcinoma. Radiotherapy (RT) may enhance systemic responses of PD-1 inhibitors and lenvatinib. This study was designed to assess the safety and feasibility of toripalimab plus lenvatinib with or without RT in advanced BTC.

**Methods:**

This study involved 88 patients with advanced BTC receiving toripalimab plus lenvatinib with or without RT from the clinical trials (NCT03892577). Propensity score matching (PSM) (1:1) analysis was used to balance potential bias. The overall survival (OS), progression-free survival (PFS), objective response rate (ORR), and adverse events (AEs) were evaluated.

**Results:**

After PSM, the final analysis included 40 patients: 20 receiving toripalimab plus lenvatinib without RT (NRT); 20 receiving toripalimab plus lenvatinib with RT. The AEs were more frequent in the RT group than in the NRT group without treatment-associated mortality. The addition of RT did not cause specific AEs. The median PFS was significantly longer with RT (10.8 versus 4.6 months, p<0.001). The median OS was 13.7 months with RT versus 9.2 months in the NRT group (p=0.008). The ORR was 35% (95% CI: 12.1-57.9) in the RT group versus 20% (95% CI: 0.8-39.2) in the NRT group.

**Conclusions:**

The addition of RT may enhance the efficacy of toripalimab plus lenvatinib. Toripalimab plus lenvatinib with RT have a good safety profile without an increase in specific toxicities in advanced BTC patients.

## Introduction

Biliary tract carcinoma (BTC), including intrahepatic cholangiocarcinoma (ICC), extrahepatic cholangiocarcinoma (ECC), and gallbladder cancer (GBC), are aggressive malignancies (1). Most patients are diagnosed at an advanced stage with a poor prognosis (2, 3). Chemotherapy has been the mainstay of treatment for patients with advanced BTC (2, 4). However, conventional chemotherapy is often accompanied by side effects and the limited survival benefit, necessitating an evaluation of alternative drug combinations (5).

PD-1/PD-L1 inhibitors have exhibited encouraging therapeutic effects. However, the response rates of either PD-1/PD-L1 inhibitors alone or PD-1/PD-L1 inhibitors with targeted therapies remain less than ideal in BTC (6, 7). Continuous exploration has been made to improve the response of PD-1/PD-L1 inhibitors, including PD-1/PD-L1 inhibitors combined with chemotherapy (8) or locoregional treatment approaches (9–11). The phase III TOPAZ-1 study showed that the combination of durvalumab plus gemcitabine and cisplatin significantly improved the survival of patients with advanced BTC (12). Recently, durvalumab plus gemcitabine and cisplatin proved as first-line treatment by FDA and NCCN guidelines. New data have emerged that radiotherapy work in synergy with immunotherapies to increase patient response (13, 14). A study showed that adding RT into the combination of PD-1/PD-L1 inhibitors and targeted therapy was feasible and could improve treatment outcomes (15). However, combination of immunotherapy plus radiotherapy may lead to more AEs. Data on immunomodulatory effects of RT in BTC remains limited.

Toripalimab, a humanized programmed death-1 (PD-1) antibody, has shown a manageable safety profile and has promising antitumor activity in patients with advanced gastric cancer and metastatic mucosal melanoma (16, 17). Toripalimab shows antitumor efficacy in cholangiocarcinoma (18).

Considering the different anti-malignancy mechanisms of lenvatinib, toripalimab, and RT, combining these three modalities may show a potential synergic effect and promising preliminary efficacy results in advanced BTC. In this study, we assessed the safety and feasibility of RT plus toripalimab and lenvatinib in patients with advanced BTC.

## Materials and methods

### Patient characteristics and matched cohorts

This retrospective study assessed the safety and feasibility of non-first-line toripalimab plus lenvatinib with RT in advanced BTC. Advanced BTC was defined as initially diagnosed unresectable BTC (histologically confirmed ECC, ICC, or GBC by biopsy or surgical specimen). Other eligibility criteria included a good physical status with an Eastern Co-operative Oncology Group (ECOG) performance status score of 0–1, Child-Pugh A or B liver function status, at least one measurable or evaluable tumor lesion according to the Response Evaluation Criteria in Solid Tumors version 1.1 (RECIST 1.1). The study protocol was compliant with the Declaration of Helsinki and was approved by the Institutional Review Board and Ethics Committee at Peking Union Medical College Hospital.

**Figure 1:**
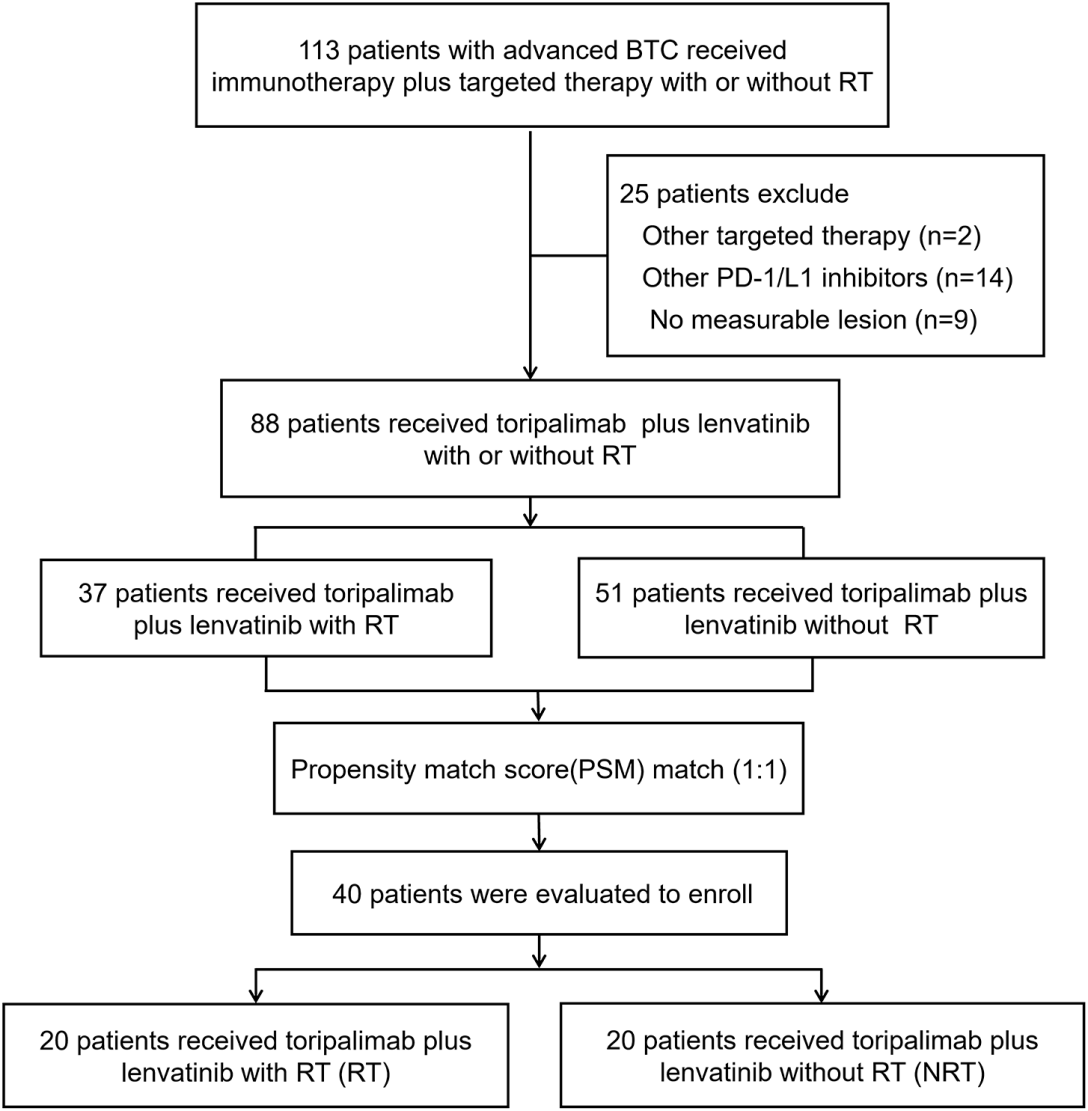
Study workflow.

A total of 113 patients were initially enrolled. Twenty-five patients have excluded: 2 patients received other target therapy; 14 patients received other PD-1/L1 inhibitors; 9 patients had no measurable lesion. Finally, 37 patients who received toripalimab plus lenvatinib with RT and 51 patients who received toripalimab plus lenvatinib without RT remained. Consecutive PSM was conducted by 1:1 matching with a caliper of 0.05 to balance potential bias. Finally, 40 patients with advanced BTC who received toripalimab plus lenvatinib with RT (RT group) or without RT (NRT group) were included for statistical analysis as a matched cohort (**Figure 1**).

### Treatment

In the NRT group, lenvatinib was administered at a dosage of 12 mg (for patients with a body weight≥60 kg) or 8 mg (for patients with a body weight <60 kg) orally once a day. The PD-1 dose included a fixed dosage of 200 mg (240 mg for toripalimab) every three weeks or 3 mg/kg every three weeks.

In the RT group, patients received intensity-modulated radiation therapy (IMRT) plus lenvatinib and toripalimab. Lenvatinib plus toripalimab was not discontinued before or after each RT session. The radiation dose was prescribed to the isocenter or 95% planning target volume as 24.0–60.0 Gy in 6-25 fractions, a single dose between 1.8 and 6.0 Gy for tumor sites at the physician’s discretion, no more than five times a week. RT was given during PD-1 inhibitors no later than six weeks (19).

### Assessments

The overall response was assessed using enhanced computed tomography (CT) or magnetic resonance imaging (MRI) according to RECIST 1.1 after the patient’s treatment. Professional radiologists evaluated the imaging examinations.

The therapeutic efficacy assessment included the objective response rate (ORR) [the percentage of patients with a confirmed complete/partial response (CR/PR)], progression-free survival (PFS) (the time from receiving toripalimab to disease progression at any site or death), the overall survival (OS) (the time from receiving toripalimab to the date of death), the disease control rate (DCR) (the proportion of patients who achieved an objective response or SD), and the safety. The adverse events (AEs) were collected and graded according to the National Cancer Institute Common Terminology Criteria for Adverse Events, version 4.0 (CTCAE 4.0).

### Statistical analysis

The Data cut-off was June 1, 2022. We performed propensity score matching (PSM) in a 1:1 fashion to further reduce selection bias. We used a caliper (i.e., the maximum distance that two cases can be apart from each other based on their estimated propensity scores) of 0.05 to prevent matches with very dissimilar estimated propensity scores. Variables used for PSM include age, sex, ECOG, subtype, and tumor stage. The Kaplan–Meier and bilateral log-rank tests were used to generate PFS and OS curves. The two treatment groups’ baseline characteristics, efficacy, and AEs were compared using the chi-square test or Fisher’s exact test. The hazard ratios of each clinicopathological feature for the OS were estimated by Cox proportional hazard modeling. All statistical analyses were undertaken using SPSS 22 (vision 22.0, SPSS, Inc., Chicago, IL) and R (version 4.0.3).

## Results

### The patient demographics and baseline characteristics

From March 19, 2019, to June 1, 2022, 40 patients with advanced BTC were included in this study: 20 in the NRT group and 20 in the RT group. The median duration of follow-up was 21.3 months. The demographics and baseline characteristics of the two groups are summarized in **Table 1**.

**Table 1:** Baseline characteristics.

Characteristics	Toripalimab plus lenvatinib with RT	Toripalimab plus lenvatinib	P-value
	(n=20)	(n=20)	
Age, years			1
≤ 65	13(65)	13(65)	
> 65	7(35)	7(35)	
Gender, n (%)			1
Male	10(50)	11(55)	
Female	10(50)	9(45)	
Tumor subtype, n (%)			0.76
Cholangiocarcinoma	14(70)	16(80)	
Gallbladder cancer	6(30)	4(20)	
ECOG performance status, n (%)			1
0	10	9	
1	10	11	
Differentiated histology, n (%)			0.08
Well	0	2(10)	
Moderately	1(5)	4(20)	
Poorly	6(30)	5(25)	
Moderately-poorly	4(20)	1(5)	
Well-moderately	0	1(5)	
Unsure	9(45)	7(35)	
Previous antitumor therapy, n (%)			
Radical surgery resection	7(35)	8(40)	1
Systemic chemotherapy	5(25)	6(30)	0.50
Targeted therapy	14(60)	14(70)	0.48
Site of metastases, n (%)			
Intrahepatic	17(85)	12(60)	0.08
Lymph nodes	18(90)	14(60)	0.12
Lung	2(10)	2(10)	1
Bone	4(20)	2(10)	0.69
Other (Uterus, adrenal glands, brain)	2(10)	1(5)	1
Radiotherapy dose (Gray)			
Median(range)	45(24-60)	–	–
Radiotherapy technique			
intensity-modulated radiation	20(100)	–	–
TNM stage, n(%)			0.33
III	10	14	
IV	10	6	
Tumor diameter, mean ± SD(cm)	4.7 ± 3.7	5.6 ± 3.7	0.90
Radiotherapy site			
Liver	14(70)	–	–
Bone	2(10)	–	–
Soft tissue or lymph nodes in the abdominal cavity	12(60)	–	–

The two groups were well-balanced regarding demographics and characteristics. The median age of the patients was 61.5 years. Cholangiocarcinoma, including ICC and ECC, is the primary tumor type (75%). Most patients had a better ECOG performance status. The two groups did not differ significantly concerning differentiated histology, previous antitumor therapy, TNM stage, tumor diameter, or sites of metastases. The pathological differentiation types of 18 patients were unknown due to a lack of further pathological tissue analyses. The liver and lymph nodes were the common metastatic sites, and other metastatic lesions included uterine metastasis (one patient) and adrenal metastases (one patient).

The radiotherapy sites were mainly distributed in the liver (70%) and soft tissue or lymph nodes (60%). The median radiation dose delivered was 45 Gy (range 24 to 60 Gy) in 6–25 fractions with IMRT. 13 (65%) patients received one course, and 7 (35%) two courses.

### Efficacy

**Figure 2:**
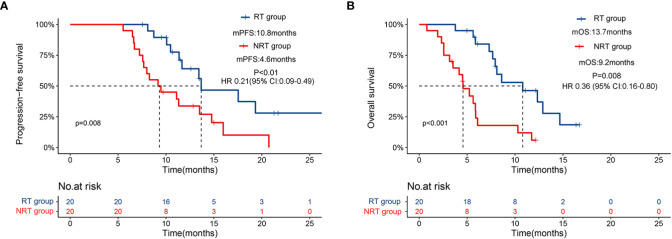
Kaplan–Meier curves for progression-free survival **(A)** and overall survival **(B)** for patients receiving PD-1 inhibitors plus lenvatinib with or without RT.

At the time of analysis, 17 patients had disease progression, and 17 patients had died in the NRT group, while 12 patients had disease progression and 10 patients had died in the RT group. The median PFS was 10.8 months (95% CI: 6.2-15.4) in the RT group versus 4.6 months (95% CI: 3.3-5.8) in the NRT group (HR 0.21 [95% CI: 0.09-0.49], p<0.01, **Figure 2A**). Likewise, the median OS was significantly longer in the RT group (13.7 months, 95% CI: 7.8-19.6) than that in the NRT group (9.2 months, 95% CI: 6.5-11.8) (HR 0.36 [95% CI: 0.16-0.80]; p=0.008, **Figure 2B**).

No patient achieved a complete response (CR) in the two groups. In the RT group, 4 patients achieved a partial response (PR), 11 patients had SD, and 5 patients exhibited progressive disease (PD) (**Table 2**). The ORR was 20% (4/20; 95% CI: 0.8-39.2), and the DCR was 75% (15/20; 95% CI: 54.2-95.8) in the NRT group. However, in the RT group, 7 patients achieved a partial response (PR), 10 patients had SD, and 3 patients exhibited progressive disease (PD), the ORR was 35% (7/20; 95% CI: 12.1-57.9), and the DCR was 85% (17/20; 95% CI: 67.9-102.1). The survival benefits in the RT group were observed. Among the two cohorts, the RT group showed a higher DCR than the NRT group but did not find a significant difference.

**Table 2:** Tumor response to treatment in each treatment group.

	Toripalimab plus lenvatinib with RT	Toripalimab plus lenvatinib	P	Effect size (95% CI)
	(n=20)	(n=20)		
Objective response rate (95% CI)	35(12.1-57.9)	20(0.8-39.2)	0.48	–
Complete response (n, %)	0	0	–	–
Partial response (n, %)	7	4	–	–
Stable disease (n, %)	10	11	–	–
Progressive disease (n, %)	3	5	–	–
DCR (n, %), 95% CI	85(67.9-102.1)	75(54.2-95.8)	0.70	–
Median progression-free survival, months (95% CI)	10.8(6.2-15.4)	4.6(3.8-5.3)	<0.01	HR:0.21(0.09-0.49)
Median overall survival, months (95% CI)	13.7(7.8-19.6)	9.2(6.5-11.8)	0.008	HR:0.36(0.16-0.80)

**Figure 3:**
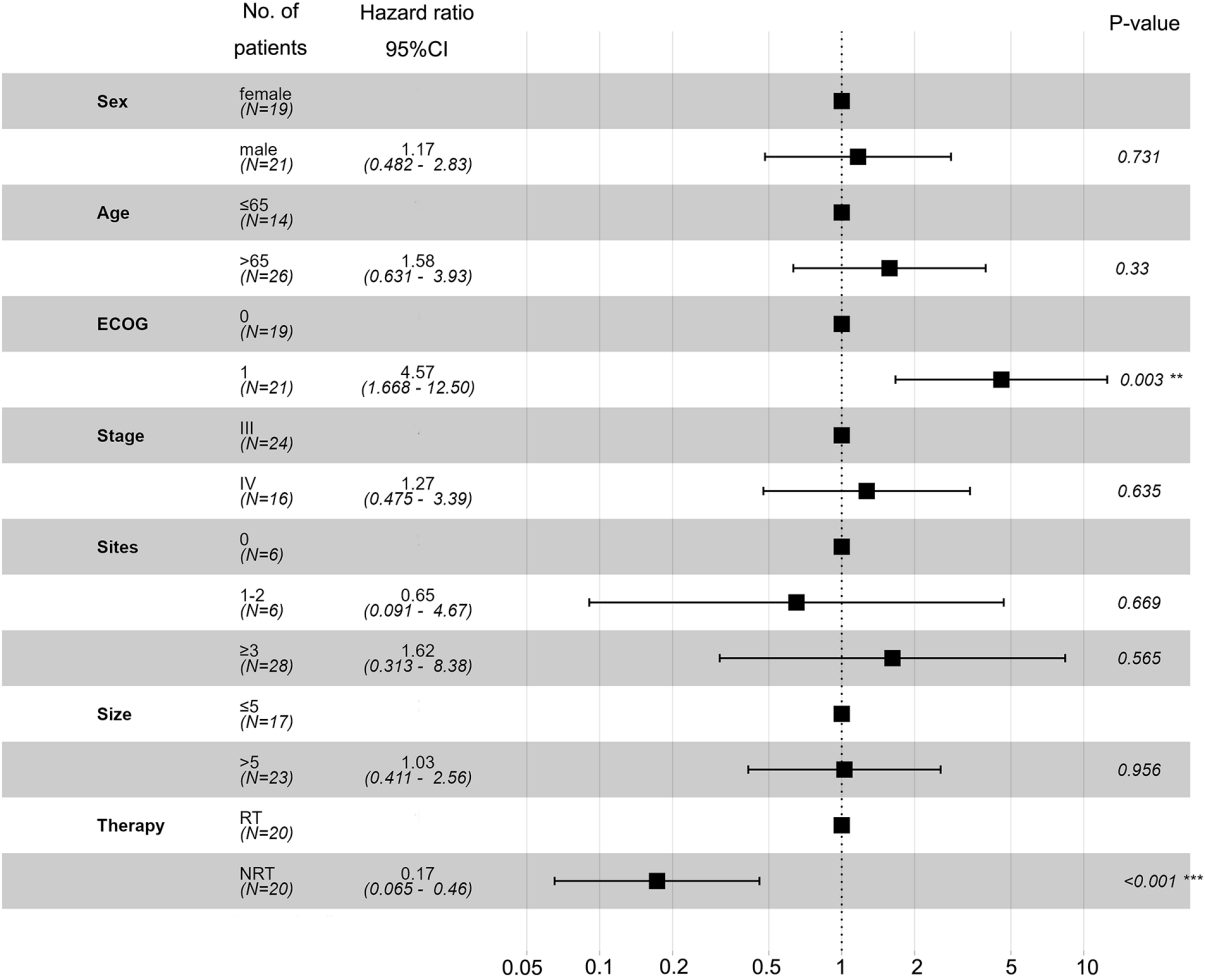
Univariate and multivariate analyses based on the Cox regression model were performed to identify independent prognostic factors associated with OS.

**Figure 4:**
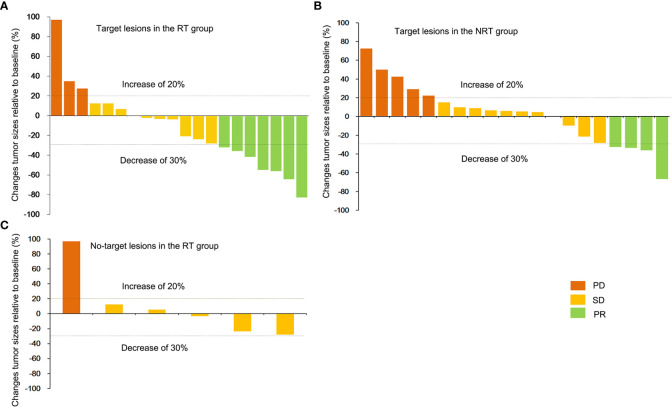
Best percentage change in the RT group. The best percentage change in the sum of the diameters of the target lesions from baseline **(A)** in the RT group and non-target lesions from baseline for nine patients in the RT group **(C)**. **(B)** shows the maximum percentage change in the sum of the diameters of the target lesions from baseline in the NRT group.

Univariate and multivariate analyses were performed to identify independent prognostic factors associated with OS. Potential predictors include age, sex, ECOG, method of treatment, and metastasis. Univariate and multivariate analyses found ECOG and treatment methods were associated with OS ***(*** **Figure 3**). **Figure 4A** shows a waterfall plot of the target lesions from baseline in the RT group: 13 of the 20 (65%) patients exhibited a decrease. In comparison, 7 of the 20 (35%) patients showed a decrease in the NRT group (**Figure 4B**). Three patients exhibited a decrease in tumor size from baseline after analysis of nine measurable non-target lesions in the RT group (**Figure 4C**).

In the RT group, one patient achieved a PR, who had been PD before radiotherapy; two patients had achieved PR, who had been SD before radiotherapy; five patients achieved SD, who was PD before radiotherapy.

### Safety

All patients experienced ≥1 adverse event (AE), and no treatment-related deaths occurred in this study (**Table 3**). The adverse events were more frequent in the RT group than in the NRT group, especially hypothyroidism [8 (5.6%) versus 1, p = 0.008]. The most common AEs (any grade) in the RT group were fatigue (70%), ALT or AST elevation (60%), and bilirubin elevation (50%), while fatigue (65%), AST or ALT increased (50%) in NRT group. The RT group had a higher incidence of grade 3–4 AEs than the NRT group. The most frequent grade 3 AEs were rash, with an incidence of 20%. One patient experienced grade 4 severe AEs (SAEs) (gastrointestinal hemorrhage). All the recorded any-grade AEs were reversible.

**Table 3:** Safety summary.

	Toripalimab plus lenvatinib with RT		Toripalimab plus lenvatinib		P-value	
	(n=20)		(n=20)			
	Any grade	Grades 3-4	Any grade	Grades 3-4	Any grade	Grades 3-4
Fatigue	14(70)	1(5)	13(65)	1(5)	0.74	1
Nausea	8(40)	2(10)	6(30)	0	0.52	0.16
Vomiting	7(35)	2(10)	4(20)	0	0.30	0.16
Proteinuria	5(25)	0	6(30)	0	0.73	–
Stomatitis	4(20)	2(10)	1(5)	0	0.16	0.16
Arthralgia	3(15)	0	1(5)	0	0.31	–
Rash	10(50)	4(20)	5(25)	1(5)	0.11	0.16
Abdominal pain	9(45)	1(5)	8(40)	0	0.76	0.33
Diarrhea	4(20)	0	5(25)	0	0.71	–
Fever	2(10)	0	1(5)	0	0.56	–
Anorexia	4(20)	0	2(10)	0	0.39	–
Gastrointestinal hemorrhage	3(15)	2(10)	2(10)	1(5)	0.64	0.56
Epistaxis	3(15)	1(5)	1(5)	0	0.31	0.33
Hypertension	9(45)	2(10)	8(40)	1(5)	0.76	0.56
Headache	3(15)	0	1(5)	0	0.31	–
Myocarditis	0	0	1(5)	0	0.33	–
AST or ALT increased	12(60)	1(5)	10(50)	1(5)	0.54	1
Bilirubin elevation	10(50)	2(10)	5(25)	2(10)	0.108	1
Hypothyroidism	8(40)	1(5)	1(5)	0	0.008	0.33
Hypoproteinemia	2(10)	0	3(15)	0	0.64	–
Thrombocytopenia	7(35)	1(5)	4(20)	0	0.3	0.33
Leukopenia	3(15)	0	4(20)	0	0.69	–

## Discussion

This is the first reported study that assessed the efficacy and safety of toripalimab plus lenvatinib with or without RT in advanced BTC patients and represents a potentially shifting approach to improve immunotherapy response. The combination of PD-1 inhibitor plus lenvatinib with RT was promising. Patients who received toripalimab plus lenvatinib with RT have significantly longer OS (13.7 versus 9.2 months, p=0.008) and PFS (10.8 versus 4.6 months, p<0.01) than patients who received toripalimab plus lenvatinib without RT. The risk of death was reduced by 64% in the RT group compared with the NRT group. Importantly, we found that toripalimab plus lenvatinib with RT were well tolerated.

In this study, patients accepting toripalimab plus lenvatinib with RT achieved approximately 35% ORR and 85% DCR, which were higher than the toripalimab plus lenvatinib regimen in our study and previous studies (7, 20, 21). The response rates of toripalimab with targeted therapies in BTC are not satisfactory. Previous studies showed that lenvatinib plus pembrolizumab has an ORR of 10% to 25% in advanced BTC (7, 20). Recently, a retrospective study of 74 patients who received PD-1 inhibitor plus lenvatinib revealed that the ORR was 20.27% (95% CI: 10.89%–29.65%), and the DCR was 71.62% (21). A pool analysis showed that pembrolizumab plus RT significantly increased responses and outcomes in patients with metastatic non-small-cell lung cancer (22). A growing body of evidence suggests that the addition of RT to PD-1 inhibitor may improve the efficacy of immune checkpoint inhibitors (ICIs) (23, 24), where RT is administered before ICIs or concurrently with ICIs (25).

The addition of RT represented an encouraging response: one patient converted from PD to PR, two patients achieved PR from SD, and five from PD to SD. In addition, we observed that both target and non-target lesions in three patients were reduced, indicating that RT may have a synergistic effect with PD-1 inhibitors and lenvatinib. Evidence has revealed that radiation can exert potent immunomodulatory effects (26). Previous studies have demonstrated that radiation could induce immunogenic cell death (ICD), release tumor antigens and promote T-cell-mediated immune response against antigens derived from dying cells (23, 27–29).

The optimal radiotherapy dose, fractionation, timing, and target selection currently lack a consensus (30, 31). To choose the optimal radiation dose and fractionated dose, on the one hand, it is necessary to ensure that antitumor immunity is fully activated. On the other hand, the occurrence of adverse reactions should be minimized. Likewise, there is no clear framework for whether RT should be performed before or after PD-1/PD-L1 inhibitors (32). The sequence of radiotherapy and immunotherapy still needs further study and comparison.

Although the incorporation of RT into immunotherapy caused more AEs, they were generally manageable. The adverse events in the RT group were consistent with previous reports: fatigue was the most common all-grade adverse event (33). One patient experienced grade 4 severe AEs (SAEs) (gastrointestinal hemorrhage). Gastrointestinal hemorrhage was controlled after drug discontinuation and active management. No death-related adverse effects occurred. The combination of RT plus non-first-line toripalimab and lenvatinib could have a good safety profile.

We acknowledge that this study has some limitations. First, as a single-center retrospective study, the interpretation of the efficacy and safety of the combination of RT plus toripalimab and lenvatinib must be very cautious. Prospective studies are needed to validate the findings further. Second, some selection biases, including recall, observation, and selection biases, arose from the limited sample size and a retrospective study. A heterogeneous population of patients cannot be ruled out. Third, this study lacks evidence of synergy between radiation and immunotherapy, such as immune cell infiltration and transcriptional changes in tumor cells before and after radiotherapy. Although the study has certain limitations, these “real” data are still helpful for prospective follow-up studies.

## Conclusions

Toripalimab plus lenvatinib with RT are safe and well tolerated in advanced BTC. Toripalimab plus lenvatinib with RT may prolong the survival of patients with previously treated advanced BTC. The addition of RT may enhance the efficacy of toripalimab and lenvatinib. Further research on prospective larger cohorts is needed.

## Data availability statement

The original contributions presented in the study are included in the article/**Supplementary Material**. Further inquiries can be directed to the corresponding authors.

## Ethics statement

The studies involving human participants were reviewed and approved by the institutional review board and ethics committee at Peking Union Medical College Hospital. The patients/participants provided their written informed consent to participate in this study.

## Author contributions

YCW, NZ, and JX collected the data and wrote the manuscript. XBY and HZ designed and examined the study. YYW, LZ, and JC helped to collect the literature and participated in discussions. CZ, JX, and YYW performed the statistical analyses. All authors contributed to the article and approved the submitted version.
