# Supplementary material for: Safety and feasibility of toripalimab plus lenvatinib with or without radiotherapy in advanced BTC
Source: Front Immunol. 2023 Jan 17;14:1084843. doi: 10.3389/fimmu.2023.1084843 (PMC9887048; doi:10.3389/fimmu.2023.1084843)
Supplement: Supplementary file 1 [file Table_1.docx]

**Table S1 Tumour response to** **all eligible patients**

|  | RT plus PD-1 inhibitors and TA (n=51) | PD-1 inhibitors plus TA  (n=37) |
| --- | --- | --- |
| Objective response rate (95% CI) | 21.6% (10-33.3%) | 29.7 % (14.3-45.2%) |
| Complete response (n, %) | 0 | 0 |
| Partial response (n, %) | 11 | 11 |
| Stable disease (n, %) | 24 | 17 |
| Progressive disease (n, %) | 8 | 4 |
| NA | 8 | 5 |
| DCR (n, %), 95% CI | 68.6(55.4-81.8%) | 75.7%(61.2-90.2%) |
| Median progression-free survival, months (95% CI) | 10(7.5-12.5) | 5(4.2-5.8) |
| Median overall survival, months (95% CI) | 13 (7.7-18.3) | 10(7.9-12.1) |
